# Knowledge of fertility and perception of fertility treatment among adults with sickle cell disease (KNOW FERTILITY)

**DOI:** 10.3389/fgwh.2023.1191064

**Published:** 2023-06-08

**Authors:** Bria Carrithers, Maidah Raja, Alison Gemmill, Kamaria C. Cayton Vaught, Mindy S. Christianson, Sophie Lanzkron, Lydia H. Pecker

**Affiliations:** ^1^ Medical College of Georgia at Augusta University, Augusta, GA, United States; ^2^ Division of Hematology, Department of Medicine, Johns Hopkins School of Medicine, Baltimore, MD, United States; ^3^ Department of Population, Family, and Reproductive Health, Johns Hopkins University Bloomberg School of Public Health, Baltimore, MD, United States; ^4^ Division of Reproductive Endocrinology & Infertility, Department of Gynecology & Obstetrics, Johns Hopkins University School of Medicine, Baltimore, MD, United States; ^5^ Division of Reproductive Endocrinology & Infertility, Johns Hopkins School of Medicine, Baltimore MD, United States

**Keywords:** sickle cell disease, fertility, infertility, knowledge, treatment

## Abstract

**Introduction:**

This study assessed fertility knowledge in adults with sickle cell disease using the Cardiff Fertility Knowledge Scale and Fertility Treatment Perception Survey and compared knowledge scores in respondents with sickle cell disease to previously reported unaffected cohorts.

**Methods:**

This cross-sectional study surveyed adults over age 18 with sickle cell disease at an adult sickle cell disease center using a 35-question survey addressing infertility risk factor knowledge and perceptions of fertility treatment. Analyses included summary statistics for continuous and categorical variables, univariate linear regression, and Mann-Whitney U tests for group comparisons of Fertility Knowledge Scale scores. Fertility Treatment Perception Survey scores were measured by medians of the two positive statements and four negative statements to generate separate positive and negative treatment belief scores. Statistical significance was set at *p* < 0.05 for all analyses.

**Results:**

Ninety-two respondents (71 female, 21 male) with median age of 32 years (IQR: 25.0, 42.5) completed the survey between October 2020-May 2021. Sixty-five percent of respondents reported taking sickle cell disease treatment and 18% reported refusing at least one sickle cell disease treatment due to fertility concerns. The mean Fertility Knowledge Score was 49% (SD: 5.2), lower than reported in an international cohort (57% vs. 49%, *p* = 0.001), and higher than in a cohort of reproductive-aged Black women in the USA (49% vs. 38%, *p* = 0.001). Less than 50% of respondents correctly identified common infertility risk factors including sexually transmitted infections, advanced age, and obesity. Mean positive fertility perception score was 3 (IQR 3, 4), and negative fertility perception score was 3.5 (IQR 3, 4). Factors associated with agreement with negative fertility perception statements included: trying to conceive, refusing sickle cell disease treatment, and undergoing fertility treatment.

**Discussion:**

Opportunities exist to improve knowledge of infertility risk factors among adults with sickle cell disease. This study raises the possibility that nearly one in five adults with sickle cell disease refuse SCD treatment or cure due to infertility concerns. Education about common infertility risks factors needs to be addressed alongside disease- and treatment- associated fertility risks.

## Introduction

Sickle cell disease (SCD) is a common, inherited autosomal recessive hematologic disorder with myriad life limiting complications (1). Improved childhood survival is leading to a growing population of adults facing SCD-specific reproductive healthcare challenges which include the accumulation of disease- and treatment-associated infertility risk factors (2–7). Women with SCD have late onset menarche, accelerated decline in ovarian reserve, and an increased risk of miscarriage (5, 8–10). Chronic pain is a risk factor for dyspareunia (11) and SCD negatively impacts sexual function, which is poorer for those with SCD than in unaffected people, a finding associated with infertility in one study (12). For men with SCD, infertility risks are a consequence of hypogonadism, sperm abnormalities, recurrent priapism, and erectile dysfunction (3, 13).

SCD treatments and cures are transformative and life-sustaining, and some jeopardize future fertility (4, 6, 7). Hematopoietic stem cell transplant and gene therapy require exposure to gonadotoxic chemotherapy agents and, sometimes, total body radiation. For those undergoing radiation, the testicles, but not the ovaries, may be shielded from the gonadotoxic effects of radiation (2). Chronic disease modifying therapies may also be gonadotoxic. Hydroxyurea is the oldest and most established SCD treatment; strong evidence exists that shows initiating treatment in childhood is contributing to increased, life-long use (6, 14). For men, hydroxyurea causes oligo- and azoospermia, an outcome that may be reversible (6, 15). In women, hydroxyurea use is associated with diminished ovarian reserve (5, 8, 16) and possibly with miscarriage in women with SCD (5, 17) but causality is not established. Even less is known about whether other chronic SCD treatments are gonadoprotective or gonadotoxic (7, 18).

Infertility risks are a patient-centered SCD concern that impact use of transformative SCD treatments and cures (18–21). A study of adolescents with SCD identified that future biological parenthood is important to affected young people and that few had received information addressing fertility in SCD (22). Little research establishes what adults with SCD know about general or SCD-specific infertility risks. Understanding the baseline fertility knowledge of people with SCD can inform counseling patients about complex and sometimes uncertain infertility risks (10, 23).

The aim of this study was to assess fertility knowledge in adults with SCD using the Cardiff Fertility Knowledge Scale (CFKS) and Fertility Treatment Perception Survey (24, 25). A secondary objective was to compare CFKS and Fertility Treatment Survey score results in adults with SCD to previously reported scores from a large international cohort and a cohort of women from Georgia, USA (24, 25). We hypothesized that adults with SCD would have lower fertility knowledge than comparator cohorts without SCD and that adults with SCD would have negative views regarding fertility treatments.

## Methods

### Study population

The Johns Hopkins Institutional Review Board approved this cross-sectional survey. We included adults with SCD aged 18 years or older and cared for at the Sickle Cell Center for Adults between October 10, 2020, and May 26, 2021. Initially we obtained oral consent during clinic visits. However, the COVID−19 pandemic impeded in-person recruitment and we subsequently recruited participants during telemedicine visits and after January 21, 2021, consented respondents received a survey link sent through the electronic medical record.

### Survey design

The survey consisted of 35 questions including the CFKS and Fertility Treatment Perception Survey, questions assessing demographics, SCD treatment use, and reproductive outcomes (Supplementary Material S1). The Flesch-Kincaid Grade Level of the survey was 5.8. Demographic characteristics collected included age, sex, and educational attainment. Information on current SCD treatment use was collected and categorized as hydroxyurea, chronic blood transfusions, voxelotor, crizanlizumab, l-glutamine, HSCT, gene therapy or no therapy. Survey questions assessed birthing or fathering a child, age in years at the first child's birth, months currently trying to conceive (<6 months, 6 to 12 months, and ≥12 months), and referral to a fertility specialist for testing. For those reporting fertility referral, we asked whether a child was conceived through fertility treatment.

The CFKS is a 13-question survey that assesses knowledge of (1) causes for reduced fertility, (2) common misconceptions about fertility, and (3) basic facts about infertility. Answer choices are true, false, or do not know. True responses are considered correct, and false or do not know answers are considered incorrect. The total survey score is reported as a percentage of correct responses.

The Fertility Perception Treatment survey is co-administered with the CFKS (24, 26) and includes two positive and four negative statements about fertility. The two positive fertility statements assess the safety and efficacy of fertility treatment. The four negative fertility statements assess fertility treatment as a scary experience, short-term effects of fertility treatment, long term effects of fertility treatment, and emotional problems related to fertility treatment. Agreement is measured using a five-point Likert scale with higher scores indicating greater agreement with each statement (1 = strongly disagree and 5 = strongly agree). The two positive statements are summed to generate a positive treatment belief score and the four negative statements are summed to generate a negative treatment belief score.

### Analysis

Summary statistics with medians (interquartile range, IQR) for continuous variables and frequencies for categorical variables are reported. Mann-Whitney *U* test with a Z-continuity correction of <0.05 was conducted to evaluate if the CFKS scores varied by demographic or reproductive characteristics and SCD treatment use. We compared CFKS scores to scores reported separately in international reproductive-aged populations (24) and in reproductive-aged, unaffected Black women cohort from Atlanta, Georgia (25). We performed univariate linear regression to determine the average difference in the CFKS scores by demographic and reproductive characteristics and SCD treatment use. Fertility Treatment Perception Survey agreement is measured by medians of the two positive statements and four negative statements to generate separate positive and negative treatment belief scores. The statistical significance was *p* < 0.05. All statistical analyses were performed using SAS software, version 9.4 (27).

## Results

Over seven months, we contacted 435 eligible patients and 92 completed surveys, among them 71 self-identified female and 21 self-identified male (21% survey response rate).

### Demographics

Respondent characteristics are in Table 1. The median age was 32 (IQR 25, 43) years, 49% had some secondary education, and 48% had a biological child and were pregnant at a median age of 22 (IQR 21, 29) years. Most respondents (65%) reported undergoing a SCD therapy, including hydroxyurea (*n* = 38) and chronic red blood cell transfusion (*n* = 17), and recently approved drugs, crizanlizumab and voxelotor (*n* = 5). Three respondents, all women, had a history of unsuccessful HSCT. Although most respondents (82%) did not refuse SCD treatment or cure due to infertility concerns, 18% reported refusing one or more SCD treatment due to fertility concerns (Table 2). This included 11 respondents who refused hydroxyurea, seven who refused curative therapy, and one each who refused chronic blood transfusions or crizanlizumab.

**Table 1:** Summary statistics (medians and frequencies) of demographic characteristics stratified by sex of the survey respondents.

	All *N* = 92	Men *N* = 21	Women *N* = 71
**Demographic characteristics**			
Age (years) Median (IQR)	32 (25, 42.5)	36 (24, 45)	31 (25, 40)
Secondary education *n* (%)	45 (49)	13 (62)	32 (45)
**SCD treatment *n* (%)**			
Hydroxyurea^a^	38 (41)	9 (43)	29 (41)
Chronic transfusion	17 (19)	3 (14)	14 (20)
Other	5 (5)	1 (5)	4 (6)
None	32 (35)	8 (38)	24 (34)
HSCT treatment discussion	21 (23)	6 (29)	15 (21)
HSCT done	3 (3)	0	3 (4)
**Fertility-related characteristics^b^**			
Given birth/fathered child	44 (44)	9 (43)	35 (49)
Age at first birth/fathered child (years), median (IQR)	22 (21, 29)	24 (24, 33)	21 (20, 28)
Trying to conceive, yes *n* (%)	6 (7)	1 (1)	5 (5)
Trying to conceive > 12 months, yes *n* (%)	3 (3)	1 (1)	2 (2)
Referral for fertility tests, yes *n* (%)	12 (13)	1 (1)	11 (12)
Fertility treatment performed, yes *n* (%)	6 (7)	0	6 (7)
Baby through fertility treatment, yes *n* (%)	1 (1)	0	1 (1)
Fertility treatment use (self or someone they know) *n* (%)	15 (16)	0	15 (16)

HSCT, hematopoietic stem cell transplant; IQR, interquartile range; SCD, sickle cell disease.

^a^ One survey respondent received hydroxyurea, and blood transfusion.

^b^ One survey respondent adopted a child, seven survey respondents had stepchild(ren).

**Table 2:** Types of SCD treatments refused due to fertility concerns. Eighteen percent (*n* = 17) of respondents refused SCD treatments due to fertility concerns; this includes two women respondents who refused multiple SCD treatments.

	Types of SCD treatments			
	Hydroxyurea^a,b^	Chronic transfusions^b^	Voxelotor and crizanlizumab^b^	Curative therapy
Total refused *n* = 17	11	1	1	7
Men *n* = 5	3	0	0	3
Women *n* = 12	8	1	1	4

^a^ One woman refused hydroxyurea, chronic transfusions, and curative therapy.

^b^ One woman refused voxelotor and crizanlizumab.

Sixteen percent of respondents reported fertility treatment experience either for themselves or someone they knew. Thirteen percent had been referred for fertility testing, including one man and eleven women. Among 12 respondents with a history of referral for fertility testing, six respondents (50%) endorsed using medical assistance to conceive, and among them, one person reported a successful conception.

### Cardiff fertility knowledge scale scores

The average CFKS score was 49% (IQR 31, 66), lower than an international cohort of men and women currently trying to conceive (49% vs. 57%, *p* = 0.001), and higher than a cohort of reproductive aged, unaffected Black women in Atlanta, Georgia in the U.S. (49% vs. 38%, *p* = 0.001). CFKS score among respondents trying to conceive (*n* = 6) was not different than in the international cohort of people trying to conceive (62% versus 57%).

Mean CFKS score was higher among respondents with secondary versus primary education (54% vs. 44%, *p* = 0.03), those referred for fertility tests compared to those not referred (69% vs 46% *p* = 0.001), among fertility treatment recipients versus non-recipients (76% vs 47%, *p* = 0.0002), and among respondents who knew someone that used a fertility treatment compared to those who did not (72% vs. 45%, *p* = 0.0002).

There was a non-significant trend towards higher mean CFKS score among those who refused SCD treatment due to fertility concerns compared to those who did not refuse SCD treatment due to fertility concerns (59% vs 47%). Mean CFKS score did not differ by sex (49% men vs. 44% women), age greater or less than 35 years (51% vs. 48%), status of trying to conceive versus not trying to conceive (58% vs. 49%), or by treatment choice (49% no treatment vs. 53% chronic red cell transfusion, 51% hydroxyurea, and 41% crizanlizumab or voxelotor).

### Individual survey items

Correct responses to individual questions varied from 32% to 79% (Table 3). Over 65% of respondents correctly identified smoking as a fertility risk for men and women, that neither erections nor presence of sperm production predict male fertility, and that female fertility decreases after age 36. Fewer than 40% of respondents correctly identified sexually transmitted infections or being overweight as infertility risk factors or that the clinical definition of infertility is failure to conceive after a year of regular sexual intercourse.

**Table 3:** Cardiff fertility knowledge survey scores in order of percentage of correct responses.^a^ survey questions displayed are derived from CFKS; true is indicated by (T) and false is indicated by (F).

Survey question^b^	All *n* = 92 *n* (%)	Men *n* = 21 *n* (%)	Women *n* = 71 *n* (%)
Mean CFKS Score (%)	49	49	44
If a man can achieve an erection, then it is an indication that he is fertile. (F)	72 (79)	17 (81)	55 (77)
Smoking decreases male fertility. (T)	65 (71)	15 (71)	50 (70)
If a man produces sperm, he is fertile. (F)	63 (69)	15 (71)	48 (68)
Smoking decreases female fertility. (T)	63 (69)	14 (66)	49 (69)
A woman is less fertile after the age of 36 years. (T)	62 (68)	15 (71)	47 (66)
Having a healthy lifestyle makes you fertile. (F)	57 (62)	9 (43)	48 (68)
About 1 in 10 couples are infertile. (T)	40 (44)	7 (33)	33 (46)
A woman who never menstruates is still fertile. (F)	35 (38)	3 (14)	32 (45)
People who have had a sexually transmitted disease are likely to have reduced fertility. (T)	33 (36)	7 (33)	26 (37)
These days a woman in her 40s has a similar chance of getting pregnant in her 30s. (F)	31 (34)	7 (33)	24 (34)
A couple would be classified as infertile if they did not achieve a pregnancy after 1 year of regular sexual intercourse (without using contraception). (T)	29 (32)	6 (29)	23 (32)
If a woman is overweight by more than 28 pounds, then she may not be able to get pregnant. (T)	23 (25)	1 (5)	22 (31)
If a man has had mumps after puberty, he is more likely to later have a fertility problem. (T)	17 (18)	4 (19)	13 (18)

^a^ This includes 100% completion of all statements among 92 participants.

^b^ Correct responses were true (T) and false (F).

### Fertility treatment perception survey

The Fertility Treatment Perception survey assesses positive perceptions of fertility treatment (FT) using two items and negative perceptions of FT using four items. Higher summed scores indicate greater agreement with positive or negative statements. The median average positive fertility perception score was 3 (IQR 3, 4) and negative fertility perception score was 3.5 (IQR 3, 4).

### Sub-group comparisons of fertility perception surveys

**Figure 1:**
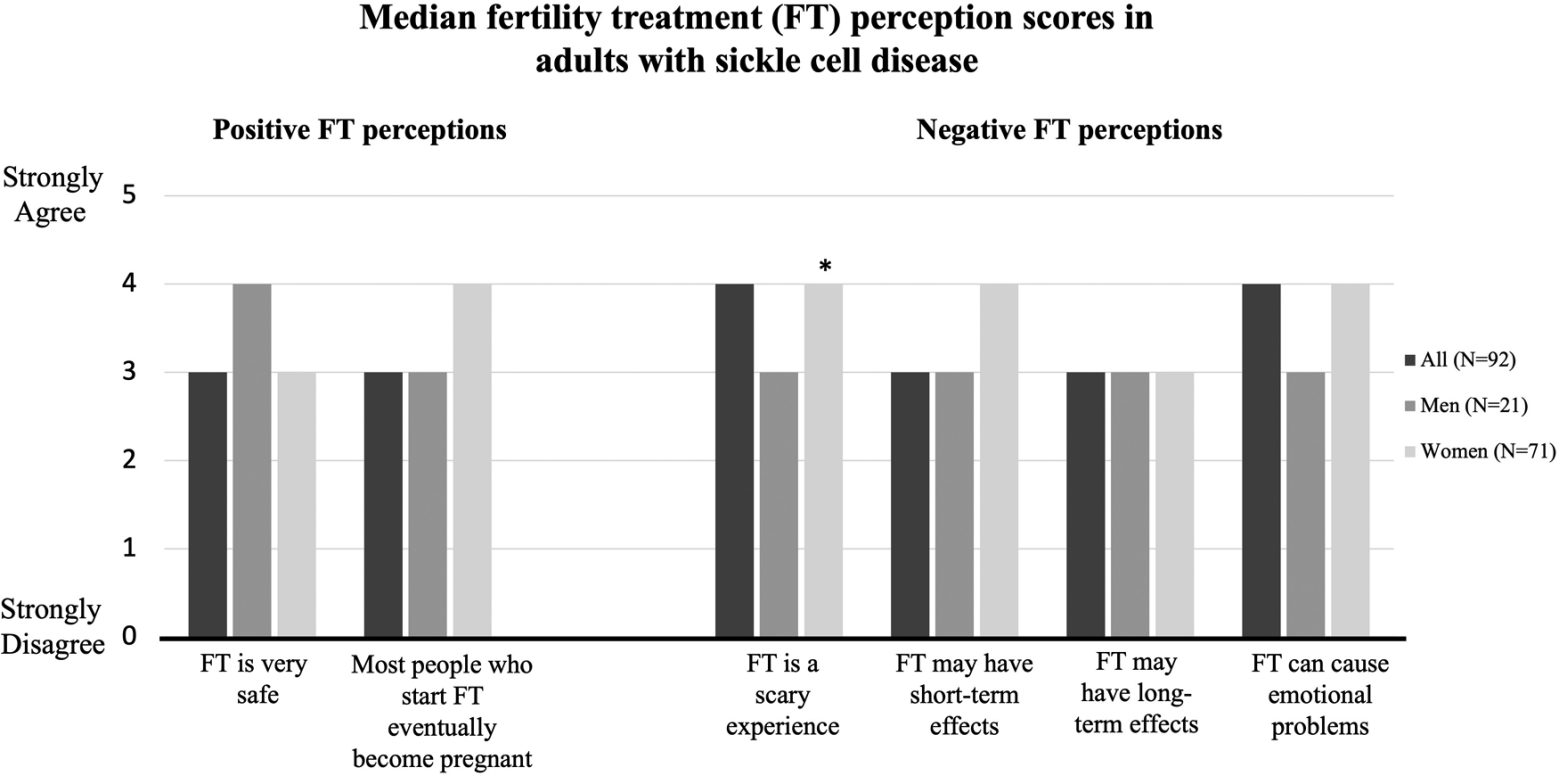
Median fertility treatment perception scores by survey respondents categorized by sex. Scores were assessed via 5-point Likert Scale (1 = strongly disagree to 5 = strongly agree). *indicates *p* < .05.

The median positive FT perception score was the same among men and women: 3.5. The median negative FT perception score was 3 for men and 4 for women. Responses to each question by all respondents and stratified by sex are shown in Figure 1. More women than men agreed with the statement “fertility treatment is a scary experience” (3 vs 4, *p* = .02).

There were several other significant differences. There were higher positive FT perception scores among respondents not taking SCD treatment (*n* = 32) compared to those taking hydroxyurea (*n* = 38) (3.5 vs. 3.0, *p* = 0.04), or to those receiving chronic transfusions (*n* = 17) (3.5 vs. 3.0, *p* = 0.03). Respondents taking hydroxyurea (*n* = 38) had higher median negative FT perception scores than those taking voxelotor, crizanlizumab, or chronic transfusions (*n* = 54) (4.0 vs. 3.0, *p* = 0.01). Also, there were higher negative FT perception scores among respondents who refused SCD treatment due to fertility concerns (*n* = 17) compared to those who did not (*n* = 75) (4.0 vs. 3.0, *p* = 0.01), respondents who were trying to conceive (*n* = 6) than those not trying to conceive (*n* = 86) (4.0 vs. 3.0, *p* = 0.005), and among respondents with a history of fertility treatment (*n* = 6) compared to those who did not undergo fertility treatment (*n* = 86) (4.0 vs. 3.0, *p* = 0.005).

There were no differences in positive FT perception scores based on SCD treatment refusal due to fertility concerns, among those trying or not trying to conceive, by age greater or less than 35 years, by education status, or by history of fertility treatment. There were no differences in negative FT perception scores by age or educational attainment.

## Discussion

In this study, adults with SCD demonstrate mixed knowledge of common infertility risks with mean CFKS scores higher than a previous study in an unaffected cohort of Black women in the U.S (26). and lower than in an international unaffected cohort (25). These results highlight an opportunity to improve fertility and SCD education for adolescents and young adults: most respondents did not identify sexually transmitted infections as an infertility risk factor or failure to conceive after a year of intercourse as the diagnosis of infertility. Although the actual number of participants attempting to conceive in the study is small (*n* = 6), 50% had been trying to conceive for over one year. Among them, only one respondent was referred for fertility testing, and had not conceived. Also, 17 respondents reported refusing at least one of the three most clearly transformational SCD interventions available, hydroxyurea, curative therapy, or transfusions, due to fertility concerns. As disease-specific fertility counseling and care interventions evolve, addressing infertility risks that affect the general population and SCD-specific risks is necessary (7, 11, 23). Counseling patients about infertility risks or infertility diagnosis creates opportunities to address fertility treatment perceptions and might affect use of fertility care (7, 23).

This study underscores the need for educational interventions to address infertility risks for adults with SCD. However, the results also suggest that fertility may be variably salient for people with SCD depending on their life-stage and SCD and fertility care experiences: people with history of fertility treatment and those trying to conceive answered more questions correctly than did those without these histories. Further, there was an overrepresentation of people with a history of failed HSCT in this sample compared to the overall clinical population at our center. This study may have been particularly appealing to people with a history of HSCT, who have established infertility risks associated with treatment (2). As care systems and treatment options continue to evolve, efforts to ensure that fertility education and care are integrated into comprehensive SCD care across the lifespan are needed (7, 28).

Here, disease experience and demographic characteristics contribute to fertility knowledge among adults with SCD. Fertility knowledge scores were not different in respondents trying to conceive versus not trying to conceive but were significantly higher in respondents undergoing fertility treatment. This expected result suggests, reassuringly, that fertility counseling can increase understanding; simultaneously a lack of knowledge among most patients highlights the need for intervention. Patient education level may be care informing: those with higher educational levels had higher fertility knowledge. In an environmental scan of education materials addressing *in vitro* fertilization with preimplantation genetic testing, the average reading level was 14.5 grade, far in excess of the 5th grade reading level advised by the U.S. Joint Commission (29). Educational interventions addressing fertility require sensitivity to the health literacy needs of individuals to eliminate disparities and ensure effective uptake of fertility knowledge (30).

In this study, fertility concerns were a reason for hydroxyurea refusal for 11 respondents and curative therapy for seven respondents. Despite overwhelming evidence of hydroxyurea's efficacy, barriers to treating children and adults persist (31, 32). There was a non-significant trend towards higher fertility knowledge among people who refused a SCD treatment due to a fertility concern. For some, hydroxyurea treatment refusal is tied to anticipation of negative side effects (33), including fertility concerns (34). Engaging patients and families about fertility care is also critical as opportunities to access curative therapies expand. The Federal Drug Administration is considering approval for gene therapy for SCD, equity concerns are raised: since gene therapy preparative regimens require use of gonadotoxic alkylating agents, access to fertility education and care are among them for the SCD community.

Here, more women reported being referred to fertility testing than men, and some women, but no men, reported knowing people undergoing fertility treatment. Our findings underscore the need for sex-specific care and suggest that well-established male infertility risk factors are inadequately addressed by existing clinical care structures. In the USA, clinicians report avoiding discussions of fertility risks with parents considering hydroxyurea for their children despite evidence that the drug is toxic to sperm in a possibly reversible manner (19). In long-term follow-up of people with SCD treated with hydroxyurea in a large Phase 4 observational cohort study, there were only 12 reported pregnancies in partners of the 441 adult men treated with hydroxyurea (6). While fertility counseling and preservation is offered to boys and men taking hydroxyurea in France, this approach has not been universally adopted (7). In the USA, there are not yet guidelines to inform fertility education, fertility preservation, or care for couples pursuing pregnancy where the partner with SCD may be male or female (28, 35, 36).

This study has limitations. We recruited patients from a single center where care is informed by an author of this study whose research focus is fertility in SCD (LHP). Additionally, selection bias is possible: this study may have attracted participation of people with fertility concerns. Those referred for fertility care, refusing treatment due to infertility concerns, and with failed HSCT may be overrepresented in this sample. The average educational attainment in this sample was high. For these reasons, possibly the CFKS scores reported in our sample are higher than the general SCD population. In addition, a small number of respondents were trying to conceive at the time of study. Fertility treatment outcomes were not obtained. In addition, both sample size and survey response rate was low, perhaps because we were forced to enroll subjects remotely due to the COVID−19 pandemic. This may reduce the generalizability to other adults with SCD, however the direction of those differences is unclear as there is limited research on this topic. The small number of participants also precluded meaningful multivariable regression analysis adjusting for sociodemographic characteristics. Further research with larger sample sizes investigating fertility knowledge, perceptions, and care is necessary. Study is also needed to compare the characteristics of people with SCD who do or do not pursue fertility care.

A strength of this study includes the use of a validated survey tool with the opportunity for comparison to other groups. In addition, this is an understudied topic: very few studies have investigated fertility knowledge, perception, or fertility care among adults with SCD (10, 18, 24). This study helps identify the type of information that needs to be incorporated into education programs for people with SCD, especially as SCD survival to reproductive years continues to improve around the globe and growing numbers of treatments or cures that potentially compromise fertility emerge.

As people with SCD may have both disease-specific and general infertility risks (7), counseling for this population will ultimately include information about advanced maternal age, STIs, smoking and increased bodyweight as potential infertility risk factors, in addition to SCD-specific risks (37). The improved survival of people with SCD, increasing use of SCD treatments and evolving landscape of curative therapies makes fertility knowledge and infertility care increasingly important for this patient population. Hopefully, the low fertility knowledge and greater negative fertility treatment perceptions among adults with SCD identified here can be addressed by integrating fertility care into the comprehensive SCD care model.

## Data Availability

The raw data supporting the conclusions of this article will be made available by the authors, without undue reservation.
