# Supplementary material for: Knowledge of fertility and perception of fertility treatment among adults with sickle cell disease (KNOW FERTILITY)
Source: Front Glob Womens Health. 2023 Jun 8;4:1191064. doi: 10.3389/fgwh.2023.1191064 (PMC10287173; doi:10.3389/fgwh.2023.1191064)
Supplement: Supplementary file 1 [file Datasheet1.pdf]

Supplement 1. KNOW FERTILITY Survey (last revised in September 2020).

Demographics

1: Sex: Male / Female
2: Age
3: What is your highest level of education? Graduate degree / Some graduate school / college / some college / high school / some high school / no high school
4: Have you ever met a bone marrow transplant doctor to discuss this treatment for sickle cell disease? Yes/No
5: Have you ever had a bone marrow transplant? Yes / No
6: Are you currently taking any sickle cell disease treatment? Yes/No
    If yes, which treatment: hydroxyurea, blood transfusions, voxelotor, crizanlizumab, l-glutamine, bone marrow transplant, gene therapy, no therapy
7: Have you or anyone you know personally ever used fertility treatments to try to have a baby or not? Yes/No
8: Have you ever refused a sickle cell disease treatment or cure because of worries that it might cause infertility? Yes / No
    If yes, which treatment: hydroxyurea, blood transfusions, voxelotor, crizanlizumab, bone marrow transplant, gene therapy

Fertility Status

1(Female, F): Have you given birth to a child?
    If yes, how old were you at the birth of your first child?
2(Male, M): Have you fathered a child?
    If yes, how old were you at the birth of your first child?
3: Have you adopted a child or children? Yes / No
4: Do you have any stepchildren? Yes / No
5(F): Are you trying to get pregnant right now?
    If yes, how long have you been trying to get pregnant? (1) < 6 mo (2) 6 – 12 mo (3) 12 – 24 mo (4) > 24mo
5(M): Is your partner trying to get pregnant right now?
    If yes, how long have you and your partner been trying to get pregnant? (1) < 6 mo (2) 6 – 12 mo (3) 12 – 24 mo (4) > 24mo
6: Have you ever been referred to a medical doctor for fertility tests or treatment? Yes/No
7 (If 6 yes, Female): Have you had treatment from a medical doctor to help you get pregnant? Yes / No
    If yes, did you have a baby with medical treatment? Yes / No
7 (If 6 yes, Male): Did you or your partner have treatment from a medical doctor to help your partner get pregnant? Yes/No
    If yes, did your partner have a baby with medical treatment? Yes/No
8 (If 6 no): Do you know anyone who has been treated by a medical doctor for help with getting pregnant? Yes/No

Cardiff Fertility Knowledge Scale (CFKS)

1. A woman is less fertile after age of 36 years. True/False/Don’t Know
2. A couple would be classified as infertile if they did not achieve a pregnancy after 1 year of regular sexual intercourse (without using contraception) True/False/Don’t Know
3. Smoking decreases female fertility. True/False/Don’t know
4. Smoking decreases male fertility. True/False/Don’t know
5. About 1 in 10 couples are infertile. True/False/Don’t know
6. If a man produces sperm, he is fertile. True/False/Don’t know
7. These days a woman in her 40s has a similar chance of getting pregnant in her 30s. True/False/Don’t know
8. Having a healthy lifestyle makes you fertile True/False/Don’t know

9. If a man has had mumps after puberty, he is more likely to later have a fertility problem. True/False/Don’t know
10. A woman who never menstruates is still fertile. True/False/Don’t know
11. If a woman is overweight by more than 28 pounds then she may not be able to get pregnant. True/False/Don’t know
12. If a man can achieve an erection, then it is an indication that he is fertile. True/False/Don’t know
13. People who have had a sexually transmitted disease are likely to have reduced fertility. True/False/Don’t know

Fertility Treatment Perceptions Survey

On a scale of 1 to 5 with 1 being strongly disagree and 5 being strongly agree rate the following statements:

1. Fertility treatment is very safe.
2. Most people who start fertility treatment eventually become pregnant.
3. Fertility treatment is a scary experience.
4. Fertility treatment may have short-term physical effects (headache, nausea).
5. Fertility treatment may have long-term physical effects (cancer).
6. Fertility treatment can cause emotional problems.
